# Rethinking balance and impartiality in journalism? How the BBC attempted and failed to change the paradigm

**DOI:** 10.1177/1464884916648094

**Published:** 2016-06-30

**Authors:** Karin Wahl-Jorgensen, Mike Berry, Iñaki Garcia-Blanco, Lucy Bennett, Jonathan Cable

**Affiliations:** Cardiff University, UK; Cardiff University, UK; Cardiff University, UK; Independent Researcher, UK; Cardiff University, UK

**Keywords:** BBC, European Union, immigration, impartiality, journalism practice, objectivity, public service broadcasting, religion

## Abstract

This article reconsiders the concepts of balance and impartiality in journalism, in the context of a quantitative content analysis of sourcing patterns in BBC news programming on radio, television and online in 2007 and 2012. Impartiality is the cornerstone of principles of public service broadcasting at the BBC and other broadcasters modelled on it. However, the article suggests that in the case of the BBC, it is principally put into practice through juxtaposing the positions of the two main political parties – Conservative and Labour. On this basis, the article develops the idea of the ‘paradigm of impartiality-as-balance.’ This paradigm prevails despite the news organisation’s commitment to representing a broader range of opinion. The paradigm of impartiality-as-balance means that only a narrow range of views and voices are heard on the most contentious and important issues. Further, it results in reporting that focuses on party-political conflict, to the detriment of a journalism which provides much-needed context.

## Introduction

This article revisits the concepts of balance and impartiality in journalism, in the context of a quantitative content analysis of sourcing patterns in BBC news programming on radio, television and online in 2007 and 2012.^1^ Impartiality is the cornerstone of principles of public service broadcasting at the BBC and other broadcasters modelled on it. However, the article suggests that in the case of the BBC, it is principally put into practice through the paradigm of impartiality as party-political balance: It is achieved by juxtaposing the positions of the two main political parties – Conservative and Labour – to the detriment of a broader range of opinion. This is the case despite the news organisation’s commitment to representing a broader range of opinion.

The article explores (a) the relationship between the dominance of elite sources and (b) a narrowing range of views and information on contentious issues and (c) an over-representation of Conservative voices. Our findings point to (d) the difficulties of moving beyond impartiality-as-balance in the context of a politically exposed public service broadcaster. This is demonstrated through an examination of sourcing patterns in BBC coverage of three complex and contentious issues – immigration, religion and Britain’s relationship to Europe.

We use the idea of a paradigm here in the sense developed by Kuhn (1962) and adapted by journalism scholars. Kuhn (1970: 42) introduced the concept of the paradigm in his work on the structure of scientific revolutions. He traced the patterns of practice and enquiry through which scientific communities define themselves and perpetuate their existence and their approach to major problems. This includes a set of shared preconceptions that underpin and shape the collection of empirical evidence, coalescing into patterns or models which overdetermine certain modes of inquiry, while excluding others. Here, we use the idea of the paradigm as it has been deployed by journalism scholars; as a model:

[That] guides those engaged in complex information-producing tasks; it focuses attention on some problems and necessarily excludes others that cannot be as easily stated using the tools supplied by the paradigm. To make sense of the world, journalists, like scientists, rely on a paradigm, which remains of value so long as it provides a useful practical guide for them and they share its underlying assumptions. (Reese, 1990: 391)

In journalism studies, the concept has been primarily deployed to describe the adherence of professional cultures to the ‘paradigm of objectivity’ (e.g. Berkowitz, 2000; Hackett, 1984; Mindich, 2000) although some have also used it to describe ‘challenger paradigms’ such as alternative or deliberative models of journalism (Hackett, 2011; Robie, 2013). The term is helpful in emphasising the discursively constituted presumptions that shape practice within professional communities (see Vogel, 2009). In the case of journalism, paradigms are made and maintained through the discursive practice of journalists as they go about their reporting and story-telling, organisational rituals and routines (e.g. Tuchman, 1972), as well as in discussions the profession has about itself. Through such practices, discourses and institutions, journalistic paradigms are contested, defended and repaired, highlighting the role of journalism as an essentially defensive profession that constantly justifies its authority in constructing our collective truths (e.g. Carlson and Lewis, 2015). David Mindich (2000: 7) discussed the idea of balance as a paradigm for objective journalism. He cited the ‘seesaw’ metaphor for journalism as having shaped journalism practice throughout the 20th century, with frequent references to covering ‘both sides’ of an issue in discussions of objectivity and impartiality. This metaphor of the seesaw is also, as we shall see, explicitly used in the BBC’s own critical engagement with impartiality.

## Theorising impartiality and balance

The idea of impartiality is central to the tenets of public service broadcasters around the world (e.g. Flood et al., 2011) and has been particularly important to the BBC. It is written into the Royal Charter which governs the BBC and guarantees its independence (e.g. Starkey, 2007: xix). According to Helen Boaden (2010), the former director of BBC news, and current director of BBC radio, ‘impartiality is in our DNA – it’s part of the BBC’s genetic make-up’. Impartiality has been taken to mean that coverage should be unbiased, balanced, objective, open-minded and avoid favouring one side over another (Cushion, 2011: 33). While impartiality is *related* to the professional practices and ideals of balance and objectivity, it is also a distinct concept:

[Impartiality] is to be distinguished from balance (the allocation of equal space to opposing views) and objectivity (by which journalists usually mean an effort to exclude subjective judgement). Impartiality involves no more than the attempt to regard different ideas, opinions, interests or individuals with detachment. (Cox, 2007, cited in Sambrook, 2012: 5)

Ensuring impartiality, then, ideally entails the provision of a broad view of the range and weight of opinion on a particular topic. It means that journalists play an active role in *constructing* the narrative surrounding the range of opinion on a particular topic, as they seek to reflect the diversity of the public(s) they represent, while at the same time attempting to ‘bind the nation and nurture a collective climate of rational opinion formation’ (Hendy, 2013: 38). Reporters who aim to be impartial should ‘take account of (i) a full *range* of views and opinions; (ii) the relative *weight* of opinion…; and (iii) changes that occur in the range and weight of opinion over time’ (Hartley, 1992: 145). This definition demonstrates that an impartial approach requires a complex set of professional assessments. This is also reflected in the BBC’s editorial guidelines, which state a commitment to reflecting a

breadth and diversity of opinion across our output as a whole, over an appropriate period, so that no significant strand of thought is knowingly unreflected or under-represented. We will be fair and open-minded when examining evidence and weighing material acts. (British Broadcasting Corporation, 2014)

Together, these statements highlight how, given the time and space constraints of broadcasting, decisions about which voices and positions to represent necessarily involves selectivity and significant curatorial responsibility on behalf of the journalist (see also Engelbert and McCurdy, 2012). It also entails a careful judgement of the different articulations of impartiality across genre and content types, including distinctions between domestic and international content (e.g. BBC Trust, 2012: 12–13). Implicit in these conceptions is a belief that, just as journalists have a ‘gut feeling’ for news (Schultz, 2007), they also have specially developed skills for picking up the range of views and voices on a particular issue, and an empirical approach to ‘examining evidence and weighing material acts’. This, however, is contradicted by research which suggests that journalism is largely an oral culture which relies on evidence gathered in face-to-face conversations with like-minded peers and friends, and takes such views to be representative of the broader public, when in fact they are often skewed by the assumptions of insider cultures (e.g. King and Schudson, 1995; Sumpter, 2000).

The BBC – along with other public service broadcasters – has been acutely aware of these issues and has strict editorial guidelines concerning the coverage of controversial issues and the inclusion of a range of views and voices (*BBC*, 2014). It has a tradition of being highly self-critical in examining its own editorial policy and content. The BBC is politically exposed because much of its funding comes from licence-fee income, directly paid by its audience. It is a frequent target for critics of the licence fee. These include voices in the right-wing press (e.g. Aitken, 2013), as well as right-leaning lobbying groups and think tanks such as the Conservative Party-funded Civitas and Newswatch, an organisation whose main aim it is to check the ‘BBC for EU bias’ (http://news-watch.co.uk/, accessed 14 July 2014).

As a result, the BBC routinely reviews its programming, with a particular interest in its application of principles of impartiality. Since 2007, the BBC Trust has commissioned a series of impartiality reviews examining the broadcaster’s coverage of a range of issues, including business and economics coverage, science, arts, rural affairs, the Arab Spring and the four nations in the United Kingdom. It is in this spirit that the Bridcut Review – a review designed to assess the state of impartiality more broadly – was carried out for the BBC Trust in 2007. It followed on from calls to rethink the concept in a more inclusive fashion. For example, in 2006, the Director of BBC Global News, Peter Horrocks, introduced the idea of ‘radical impartiality’, in stressing ‘the need to hear the widest range of views – all sides of the story’ to ensure that members of the public feel respected and represented in news coverage (Horrocks, 2006).

Horrocks’ position reflected a broader concern with the prevailing paradigm through which impartiality is practised, based on the recognition that it has tended to result in the representation of a limited range of views and voices. The Bridcut Review expanded on this, and suggested what would amount to a contestation of the paradigm of impartiality-as-balance: Lord Bridcut suggested that we need to include more than two sides to an argument, moving on from a ‘seesaw’ model, where impartiality was understood as the representation of polar opposites. Instead, he proposed a ‘wagon wheel’ model of impartiality:

Impartiality today requires a greater subtlety in covering and counterpointing the varied shades of opinion – and arguably always should have done. Whereas opinion used to be balanced in simple alternatives – and could be measured in tilts of the seesaw or swings of the pendulum – nowadays a more appropriate metaphor might be the many spokes of the wagon wheel … One opinion is not necessarily the exact opposite of another, nor do they all reach the extremity of available argument. (BBC Trust, 2007)

The recommendations of the Bridcut Review were subsequently incorporated into the BBC editorial policy. The data used in this article were originally commissioned as part of the BBC Trust’s impartiality review on the breadth of opinion – one of a series of regular reviews of the broadcaster’s performance (Wahl-Jorgensen et al., 2013). What we were asked to investigate was whether this editorial policy change and commitment to a new understanding of impartiality had actually translated into practice between 2007 and 2012. Concretely, we were asked to study whether there has been a shift away from what the Bridcut review referred to as a ‘seesaw’ and towards a ‘wagon wheel’ model of impartiality, as evidenced through the presence of a broader range of opinion and voices in their broadcasting on contentious issues.

This article draws on the data gathered for this research project, using it as a case study of a high-profile news organisation trying to move towards the representation of a greater breadth of opinion. The article will chart some of the systemic difficulties of doing so, and thereby demonstrate broader limitations around how ideals of impartiality are put into practice. In particular, as the article will suggest, impartiality has tended to be understood and practised with reference to creating ‘balance’ by giving equal time to opposing political views (Maras, 2013: 61) – what we refer to as the paradigm of impartiality-as-balance. What this means, in practice, is that if a journalist uses a politician from the governing political party as a source for a story, they are also required to quote a member of the opposition party to avoid the appearance of bias on the issue. Similarly, if journalists are covering a controversial issue, it is customary to quote at least two opposing viewpoints. This is problematic in terms of the epistemology of journalism or the system through which its claims to knowledge are made, because it may be that not all sides are equal, or there are more than two sides to a story (Boyce, 2007; Starkey, 2007: xix). It steers journalistic story-telling in the direction of conflict and controversy, when in fact there may be a consensus – as in the case of coverage of scientific debates such as that over climate change, or the safety of the Measles, Mumps and Rubella (MMR) vaccine (see also Starkey, 2007: 38). What we here refer to as the paradigm of impartiality-as-balance has been criticised for simplifying the political spectrum and narrowing the range of views on contentious topics – a position also reflected in the Bridcut Report’s (BBC Trust, 2007) metaphor of the wagon wheel.

As this article will demonstrate, the paradigm of impartiality-as-balance has the consequence of reinforcing an institutionalised preference for official and elite sources. This is not a new finding, but one which is well-established in the literature on journalistic sourcing, starting with Gans’ (1979) classical newsroom sociology study, *Deciding What’s News*, which showed that ‘Knowns’ (political, social and economic elites) appear four times as often in the news as ‘Unknowns’ (ordinary people; including victims and protesters). This pattern of elite dominance has been confirmed in subsequent research. Because journalists overwhelmingly rely on the voices of elites (e.g. Manning, 2001), they have a disproportionate influence on the media agenda (Reese, 1990), acting as the ‘primary definers’ who set the framework of interpretation against which all subsequent voices are forced to insert themselves (Hall et al., 1978). By contrast, ordinary people who appear in the news are constructed primarily as passive consumers, reacting to the agendas set by these elites (Lewis et al., 2005). This means that journalism reproduces the power structure of the society (e.g. Berkowitz, 2009: 109; Carlson and Franklin, 2011: 1). As Charles Hendy (2013) pointed out, being ‘detached avoids overt partisanship; but it can also end up with a narrow range of voices conveying establishment values’ (p. 30). The systematic over-representation of elite views, and their central role in the media agenda, is a particularly salient concern for a public service broadcaster like the BBC, which is committed to reflecting not just the activities and views of elites, but of the society as whole.

This study, then, examines how a major news organisation committed to representing a broad range of views has sought – and ultimately failed – to address the systemic problems brought about by the paradigm of impartiality-as-balance. More concretely, the article establishes (a) the relationship between the dominance of elite sources and (b) a narrowing range of views and information on contentious issues and (c) an over-representation of Conservative voices. Our findings point to (d) the difficulties of moving beyond impartiality-as-balance in the context of a politically exposed public service broadcaster.

## Methodology

This article is based on a quantitative content analysis of hundreds of news items and thousands of sources, comparing the breadth of opinion of the BBC coverage in 2007 and 2012. Content analysis is commonly used as a method for examining journalistic sources (e.g. Riff et al., 2014: 1), as it enables researchers to generate quantitative data describing larger samples. The content analysis reported here focuses on sourcing patterns in the coverage of three topics: immigration, religion in the United Kingdom and UK’s relationship to Europe, in October and November 2007 and 2012, examining a range of news and current affairs programming, as well as online stories from the BBC News website. The three topics were selected in consultation with the BBC Trust. While this article does not engage with the substance of these topics in detail, it is worthwhile noting that these are particularly interesting as examples of complex and contentious issues which, in the view of the BBC itself, require the representation of a broad range of opinions (see also BBC Trust, 2007: 7). This is necessary to adequately represent the diversity of viewpoints – particularly significant on the most controversial of issues but a broader priority for the BBC as discussed above.

To investigate any changes in the breadth of opinion over time, we studied weekday coverage of these topics over a month-long period between 15 October and 15 November in 2007 and 2012, respectively. Our research team examined thousands of news reports on television and radio, but coded only stories relevant to the three topics of religion, immigration and Britain’s relationship to Europe. Over the sample period, we identified a total of 254 stories on these topics on broadcast platforms, out of which 85 appeared on television programmes and 169 on radio. Furthermore, we identified 246 relevant online stories from the BBC News website. Altogether, the study reported here is based on the analysis of 500 stories. Our coding scheme for this study was constructed to provide a quantitative description of whose voices were heard. The detailed examination of source types allowed us to investigate whether the range of voices has shifted between 2007 and 2012. We examined up to 16 sources in each story, for a total of 2165 sources in the sample overall.^2^ Although the story was the unit of analysis, this was essentially a sourcing study, allowing for a detailed picture of sources, including an examination of what types of actors they were and their political orientation.^3^ This approach enabled us to measure the diversity of sources, which gives some indication of whether programming included a range of voices – incorporating a ‘wagon wheel’ rather than a ‘seesaw’ approach. However, a diversity of voices does not necessarily translate directly into a diversity of views, and to help us assess the latter, we also carried out qualitative examinations of selected content. Further, we coded for duration of direct quotations from sources in broadcasts, as well as word counts for online content.^4^ Coding was carried out by a team of 10 experienced coders. The coding scheme was extensively piloted prior to the conduct of the content analysis by the research team. While many of the measures were adapted from previous studies (including impartiality reviews), our source type variables were refined specifically for this study through the piloting process. Intercoder reliability tests were carried out on 5 percent of the sample by two reliability coders who were part of the research team, using intercoder agreement as the measure. For all variables discussed here, the tests resulted in agreement above 80 percent.^5^ The study discussed in this article is one of two that we carried out as part of the impartiality review. A second study examined routine coverage over the same time periods, comparing BBC with its main terrestrial broadcaster competitors, Independent Television (ITV) and Channel 4 and is briefly referenced later in the article.

On television, we examined BBC News at Ten (BBC One), BBC Breakfast 7–8 a.m. (BBC One) and Newsnight (BBC Two). On radio, we coded the Today programme from 7 to 8.30 a.m. (Radio 4), Newsbeat at 12.45 p.m. (Radio 1), and 5 Live Breakfast, Your Call 9–10 a.m. (Radio 5 Live). The sample was chosen, first of all, to include a mixture of broadcast and online coverages. Second, we wanted to measure the breadth of opinion across a range of programming, including flagship programmes such as the Today programme, Newsnight and BBC News at Ten, as well as ‘softer’ news programmes such as BBC Breakfast and audience participation on Your Call. The following table displays the number of stories focusing on these topics across the programmes and platforms that we coded.

As Table 1 demonstrates, online coverage on the three topics accounted for the largest number of stories in the sample. This is not surprising given that there are fewer constraints on space and quantity of reports in the online environment. The second-most prominent programme was Today, Radio 4’s flagship morning news programme. This may be explained by the length of the programme coded in the study – an hour and a half a day, compared to 30 minutes to an hour for most of the other programmes. But it also relates to the programme format, featuring a large number of different stories, compared to Newsnight – the only other BBC programme of comparable length – which provides in-depth coverage of a few stories.

**Table 1.** Number of stories by programme and platform.

Platform and programme	Number of stories	Percentage of overall sample (%)
Online – BBC News website	246	49.2
Radio	169	33.8
Today (7–8.30 a.m.) Radio 4	137	27.4
Breakfast, Your Call (9–10 a.m.) Radio 5 Live	22	4.4
Newsbeat (12.45 p.m.) Radio 1	10	2.0
TV	85	17.0
BBC News at Ten BBC One	32	6.4
BBC Breakfast (7–8 a.m.) BBC One	33	6.6
Newsnight BBC Two	20	4.0
Total	500	100

Our sample inevitably delivers ‘snapshots’ that provide a partial view of the breadth of opinion in BBC programming. First, the sample represents a fraction of news and current affairs programming, and second, it does so over a limited and specific period of time. Although we deliberately selected time frames that avoided major news events (e.g. elections, disasters and scandals) which might have skewed the sample, it is nonetheless inevitable that certain stories and sources were particularly prominent, while others might be entirely absent. While our findings may not represent the exact distribution of sources and stories in the BBC news provision in general, they are an accurate reflection of what occurred in this particular time span in coverage of the selected topic, and indicative of broader patterns.

## Findings

Overall, our findings demonstrate little change between 2007 and 2012, highlighting the continued predominance of an understanding of impartiality in terms of balancing opposing party-political voices. Relating to this, our research shows a focus on party-political infighting rather than the substance or context of these issues. Across the three topics, the number of stories breaks down as follows (Table 2).

**Table 2.** Story topic by year.

	2007	2012	Total
Religion	83 (31.1%)	60 (25.8%)	143
Immigration	93 (34.8%)	56 (24.0%)	149
EU	91 (34.1%)	117 (50.2%)	208
Total	267 (100%)	233 (100%)	500

EU: European Union.

There was a larger number of stories on religion and immigration in 2007 than in 2012, while the European Union (EU) appeared to be a more salient topic in 2012 than it was in 2007, and accounted for the most stories overall – just over two-fifths of our entire sample. This distribution of stories does not necessarily indicate a shift in the editorial priorities of the BBC, but rather reflects the prominence of specific news events during the sample period which related to the three topics. So, for example, during October and November 2007, the Lisbon Treaty was being negotiated and political infighting among Labour and Conservative parties was central to the news agenda, and therefore featured in many of the stories about the UK’s relationship to the EU. During the same period, questions around the relationship between Radical Islam and terrorism were prominent in the religion sample. In 2012, by contrast, the most salient news events in the EU sample included tensions between the two main parties in discussions over the EU budget, while a large number of the religion stories focused on debates within the Church of England over the appointment of the new Archbishop of Canterbury. In our immigration sample, the most prominent sets of stories in each of the 2 years focused once again on political infighting – in 2007, over the employment of illegal immigrants at the Home Office, and in 2012, over the backlog in the processes of asylum cases. These were framed in terms of tensions between the two main parties. The over-riding impression left by the coverage is that what matters in mediated discussions over immigration, religion and the EU is not the broader social, political and cultural debates regarding these issues, but rather the ways in which they articulate and dramatise the perpetual battle between the two main parties.

Table 3 shows the overall distribution of source types across the sample in each of the 2 years.

**Table 3.** Overall distribution of source type by year.^a^

Source type	2007	2012	Total
Political sources (including politicians and spokespersons)	582 (49.4%)	541 (54.8%)	1123 (51.9%)
Member of the public	133 (11.3%)	85 (8.6%)	218 (10.1%)
Media/journalists	85 (7.2%)	74 (7.5%)	159 (7.3%)
Public sector	76 (6.5%)	26 (2.6%)	102 (4.7%)
Religious leader	59 (5%)	79 (8.0%)	138 (6.4%)
NGOs/activists/charities/pressure group	44 (3.7%)	43 (4.4%)	87 (4.0%)
Academic/expert/science/tech/medical	33 (2.8%)	22 (2.2%)	55 (2.5%)
Judiciary/legal	26 (2.2%)	38 (3.9%)	64 (3.0%)
Think tank	22 (1.9%)	11 (1.1%)	33 (1.5%)
Business/private company/economy	17 (1.4%)	18 (1.8%)	35 (1.6%)
Trade union	16 (1.4%)	0 (0%)	16 (0.8%)
Military	2 (0.2%)	0 (0%)	2 (0.1%)
Other	83 (7.0%)	50 (5.1%)	133 (6.1%)
Total	1178 (100%)	987 (100%)	2165 (100%)

NGO: non-governmental organisation.

a Column percent totals may sometimes be just above or beyond 100 percent due to rounding error.

The most striking finding was the dominance of political sources: they accounted for almost half of all source appearances in 2007 and more than half in 2012. On the topic of Britain’s relationship to Europe alone, political sources accounted for more than three in five (65%) of source appearances in 2007, and four in five (79.2%) in 2012. They were used almost 10 times as frequently as the second-largest source category – media and journalist sources. The EU is frequently constructed as a remote and elite-focused political institution with little relevance to the public (Dahl, 1994; Garcia-Blanco and Wahl-Jorgensen, 2013). There has long been discussions among scholars, pundits and officials about the ‘democratic deficit’ in the EU – or the extent to which the political union’s institutions fail to adequately represent its citizens (e.g. Ward, 2002). This bias is reproduced in the coverage, which focuses on debates between Eurosceptic and pro-European political voices, largely from the two main parties.

The pattern of political source dominance indicates that far from adopting a new paradigm of impartiality as a result of the Bridcut Review (BBC Trust, 2007) and editorial policy change, the tendency towards an elite and relatively narrow range of debate only intensified between the 2 years. This is perhaps not surprising in the light of the literature on journalistic sourcing discussed above. Nor is it unique to the BBC: In a second study carried out as part of the same review, where we examined national programming across the BBC, Channel 4 and ITV, there was a general pattern of dominance of party-political sources (Wahl-Jorgensen et al., 2013).^6^ But is a striking finding nonetheless, given the official editorial commitment to a different approach. It demonstrates that the paradigm of impartiality-as-balance is so ingrained within journalistic routines that it appears impossible to overcome through top-down policy changes.

Media or journalist sources made up 7 percent of sources in both years; more than any other profession aside from politicians. This number was primarily accounted for by the use of BBC specialist correspondents or editors as commentators, and by references to reports from other media. This reflects the rise of journalists used as experts – a trend also discerned by other scholars (e.g. Barnhurst, 2015). This development has coincided with, and is closely related to, the rise of interpretation in news journalism, particularly in broadcast genres (Cushion, 2011).

It is also illustrative to consider the relative salience of different sources types. Our study included up to 16 sources for each story. If we compare the presence of politicians and members of the public in terms of their prominence within the stories, a clear pattern emerges: whereas politicians account for 52.9 percent of the first eight sources, and members of the public just 8.2 percent, the pattern for the last eight sources is very different. Here, political sources make up 42.1 percent, whereas members of the public increase to 27.7 percent. This indicates that members of the public tend to appear much later in news stories than official sources, rarely contributing to shaping the lens through which news events are framed.

By contrast to politicians, members of the public were used as sources a total of 133 times in 2007 (11.4%), and 85 times in 2012 (8.5%). These often appeared late in a story, as when a story on extremist claims in a mosque booklet included a quote from a worshipper who said ‘I would be very surprised to hear that any kind of extremism or terrorist-related activities were held in this mosque, it’s not possible, it’s a very open mosque’ (http://news.bbc.co.uk/1/hi/scotland/edinburgh_and_east/7068809.stm; accessed May 12, 2016).

As such, non-elite sources were not primarily setting the agenda for debate, but *reacting* to unfolding news events. This is consistent with research on media representations of citizens which demonstrates that although ordinary people appear frequently in the news, this does not mean that they frame public debate or provide new perspectives on political issues (Lewis et al., 2005). Likewise, members of the public were far more likely to feature in brief sound bites, with the vast majority of their appearances being 1–10 seconds in length, whereas sound bites for political sources, as well as other authoritative source types such as business owners, trade union leaders, academics, police and journalists were most frequently 11–20 seconds long.

Political sources were much more likely than other source types to be featured in the opening sections of news reports. This had the consequence of framing reports from party-political perspectives which other sources then had to respond to – demonstrating the power of political sources to serve as ‘primary definers’ (Hall et al., 1978). If we look in detail at the most frequently cited political source types, there is a clear pattern: Westminster sources are by far the most prominent voices heard in BBC coverage, and the incumbent government outranks the opposition, demonstrating the persistence of the ‘Westminster bubble’ (e.g. Deacon et al., 2006) and the advantages enjoyed by incumbents in broadcast coverage (Prior, 2006). This pattern contravenes the aim to go beyond the realms of parliamentary politics articulated in the Bridcut review: ‘While continuing a thorough and conscientious job of reporting both Houses of Parliament, and the other democratic institutions within the UK, the BBC should not always feel beholden to the parliamentary model’ (British Broadcasting Corporation Trust, 2007: 35).

As a group – based on adding up *all* the references to sources in this category – government ministers and members of Cabinet topped the chart of source types. Among this group, the most frequently quoted individual in 2007 was Foreign Secretary David Miliband, who appeared 13 times (1.1%), whereas in 2012, Home Secretary Theresa May accounted for 15 source citations (1.5%). The Prime Minister was the most important *individual* source in both years, far outranking anyone else, even if the opposition leader was also highly prominent across both years. This points to the increasing personalization and presidentialization of politics in the United Kingdom (Langer, 2011) and demonstrates that it is more difficult for groups and individuals outside of the main political parties to get a voice. But it also shows that the range and diversity of views *within* each of the main political parties will tend to be under-represented, consistent with political parties’ strategy of centrally ‘controlling the message’ (e.g. Gaber, 2013).

Table 4 below looks at the prevalence of particular political source types.

**Table 4.** Most prominent types of political sources, by year.^a^

	2007	2012
Single individuals		
Prime Minister	46 (3.9%)	53 (5.3%)
Leader of the opposition	27 (2.3%)	15 (1.5%)
Groups		
Government Cabinet and Ministers	90 (7.7%)	67 (6.7%)
Members of Parliament	67 (5.7%)	95 (9.5%)
Shadow Cabinet and Ministers	46 (3.9%)	15 (1.5%)
Labels		
‘Conservatives’	28 (2.4%)	18 (1.8%)
‘Government’	26 (2.2%)	35 (3.5%)
‘Labour’	7 (0.6%)	22 (2.2%)
‘Lib Dems’	7 (0.6%)	1 (0.1%)

a These include both direct speech sources, and ones that are quoted or referred to. The percentages are of all sources in each year.

The table allows us to examine, first of all, how frequently the incumbent Prime Minister and Leader of the Opposition were used as sources. In both years, the Prime Minister was the most newsworthy single source: in 2007, Gordon Brown was quoted 46 times (3.9% of all sources in 2007) whereas in 2012, David Cameron made an appearance 53 times (5.3% of sources in 2012). Opposition politicians were, perhaps not surprisingly, less prominent than the incumbents. In 2007, there were an almost equal number of references to ‘Conservatives’ and ‘government’ (28 and 26, or 2.4% and 2.2%). By contrast, in 2012, ‘government’ was referred to 35 times (3.5%), and ‘Labour’ just 22 times (2.2%). The prevalence of sources representing the Shadow Cabinet and Ministers went down from 3.9 percent of all sources under David Cameron’s opposition leadership in 2007 to 1.5 percent under Ed Miliband in 2012. This suggests that Cameron was a more newsworthy opposition leader than Miliband, and that his government has been more successful in attracting coverage than the preceding Labour government. This may not be due to any bias on the part of the BBC, as David Cameron has widely been characterised as a more compelling media persona than Labour leaders Gordon Brown and Ed Miliband. Cameron has been seen as a new type of politician who has been spectacularly successful in ‘generating external, individualized media meta-capital and, consequently, achieving electoral success’ (Davis and Seymour, 2010: 755). As Langer (2010) points out, Cameron’s success demonstrates that

The capacity to offer a ‘human’ persona is often considered a prerequisite of political and electoral success and a key marker of contemporary leadership potential. David Cameron’s popularity and Gordon Brown’s woes in the opinion polls have often been discussed in these terms, the former’s heir-to-Blair youthful vitality and emotional openness contrasted favorably to the detached and dour Brown. (p. 61)

The pattern of Conservative dominance was also highlighted in the analysis of references to sources for which a political affiliation could be determined, as shown in Table 5 below.

**Table 5.** Political affiliation of sources, by year.^a^

	2007	2012
Conservative	123 (41.0%)	136 (48.4%)
Labour	135 (45.0%)	74 (26.3%)
Liberal Democrat	27 (9.0%)	17 (6.0%)
Conservative/Liberal Democrat coalition	0 (0.0%)	14 (5.0%)
UK Independence Party	8 (2.7%)	4 (1.4%)
Scottish National Party	0 (0.0%)	24 (8.5%)
Other	7 (2.3%)	12 (4.3%)
Total	300 (100%)	281 (100%)

a These include both direct speech sources, and ones that are quoted or referred to. The percentages are of all sources in each year.

As this table demonstrates, the Conservative Party has consistently accounted for between two in five to almost half of all sources with clear political affiliations, whereas Labour went down significantly between the 2 years – from almost half to just over a quarter. The compelling persona of David Cameron notwithstanding, this offers some evidence of editorial decision-making in favour of Conservative voices and views. As our research on the coverage of Britain’s relationship to Europe also demonstrated, there was a tendency to over-represent Conservative and Eurosceptic views, whereas the case *for* Europe was rarely heard in the BBC coverage (Berry, 2013). There may be several reasons for the apparent pro-Conservative bias in BBC coverage on the topics we examined – a finding which flies in the face of criticisms of the BBC for its perceived left-wing bias. After the publication of our report (Wahl-Jorgensen et al., 2013) which occasioned significant public debate, the BBC’s financial editor, Robert Peston, has been one among several commentators backing up the findings on the basis of newsroom experience. Following his delivery of the Charlie Wheeler lecture, he suggested that ‘the BBC’s routinely so anxious about being accused of being left-wing, it quite often veers in what you might call a very pro-establishment, [a] rather right-wing direction, so that it’s not accused of that’ (Sommers, 2014). He further argued that

[The] broadcaster had grown ‘completely obsessed’ with following the news agenda set by coverage of the right-wing papers such as *The Daily Telegraph* and *Daily Mail.* There’s a slightly ‘safety first’ thing at the BBC – that if we think the Mail or the Telegraph is gonna lead with it, then we should lead with it, ‘he said’. I happen to think that’s mad. (Sommers, 2014)

These views are consistent with previous research on UK broadcasters’ news agenda (Hargreaves and Thomas, 2002) and were echoed in informal responses to our research from other journalists, who argued that a bias towards Conservative sources may be explained by the defensiveness of the BBC, caused by constant accusations of a left-wing bias. As one anonymous BBC journalist put it in a Twitter Direct Message (DM) responding to our research findings, ‘Four of the five BBC editors I worked for were acutely conscious of the perception that the BBC is left wing and programmed to counter it’ (Anonymous, 14 February 2014). It points to a paradoxical and little recognised danger facing public service broadcasters which, like the BBC, are funded on a licence-fee model: They are, in fact, highly politically exposed and subjected to far more scrutiny – particularly with respect to any perceived political bias – than commercial media organisations.

Taken together, the two main parties accounted for almost nine in ten party-political sources in 2007, going down to just over three in four in 2012. Other party affiliations which appeared in much smaller numbers included Plaid Cymru, Democratic Unionist Party (DUP), the British National Party (BNP), Sinn Fein, as well as Independents. These, however, were almost absent from coverage on the three topics.^7^ The reasons why the two main political parties so dominated coverage do not necessarily have much to do with any deliberate neglect of minority voices, but more with the entrenched paradigm of impartiality-as-balance, combined with an institutionalised understanding of the newsworthiness of elite sources. The ways in which the paradigm limits the range of views and sources presented on each story is helpfully illustrated by examining one example in more detail – the story that contained the largest number of sources across the 2007 online sample on the topic of immigration, and therefore one that could be expected to display a diverse range of voices. The story, ‘Migration “causes pressure in UK”’ (*BBC News*, 2007), published on 17 October 2007, was occasioned by the publication of a report on the economic and social consequences of migration, and is interesting to examine for the relative prominence given to particular sources and views.

The story opened with a brief summary of the report’s findings and went on to cite the statements of ministers on the ‘clear benefits’ of migration to the British economy. It subsequently discussed the findings of the report in more detail, based on the experiences from different regions in the United Kingdom, some of them reporting concern about the adverse effects of immigration on crime, health and education. The first directly quoted source in the story was Immigration Minister Liam Byrne, who ‘said it was important to “strike a new balance” in immigration policy’:

“That means looking at the wider benefits to the British economy on the one hand, but it means we have to take into account the wider impact on British public services and life as well.”

The second source was Damian Green, shadow minister for immigration, who expressed the need for migration quotas:

“We say of course you should look at the economics, at the effects on public services, on demand for housing, school places and so on, and that then the government should set an explicit limit every year,” he said.

The pattern of according most prominence to a source representing government, immediately followed by a member of the opposition holds true across our sample. Subsequent sources in the story included Dhananjayan Sriskandarajah from the centre-left Institute of Public Policy Research, providing an analytical perspective:

“It is clear that migration brings huge economic benefits to the UK,” said Dr Sriskandarajah.

“It is also clear that, although recent migration is presenting new challenges in areas which have received large numbers of newcomers, most local communities around the country are coping very well.”

This position was counter-balanced by Sir Andrew Green, chairman of the anti-immigration pressure group, Migrationwatch UK, who ‘said migration on the scale Britain was currently facing was having a “huge impact” with “little economic justification”’;

This example shows how sources were carefully balanced in reporting – but in such a way that reporting presented each topic as one on which there were arguments *for* and *against*, rather than a broader range of views. The non-party-political voices included here to introduce a breadth of opinion come from think tanks – widely viewed as integral parts of a ‘policy elite’ (Smith, 1993) which are frequently directly linked to political interests, rather than being the voices of an independent civil society (e.g. Dinan and Miller, 2007).

Despite the dominance of elite, party-political voices in flagship programming, there was also some indication that online and phone-in platforms were more likely to include non-elite voices, representing a broader range of views. For example, genres which included audience participation – particularly the phone-in programme, Your Call and online news – were more likely to include a broad range of sources, representing opinions that were not heard elsewhere, including more extreme views. For example, in stories on religion, 23 members of the public appeared in Your Call and 40 online, accounting for 70 percent of all instances where members of the public appeared in the religion sample.^8^ However, just as there may be a hierarchy of access to the media, there is also a hierarchy of platforms – as our research shows, ‘flagship’ programmes or more prestigious platforms are less likely to include a broader range of voices and views. It does, nonetheless, suggest that alternative platforms and formats are more likely to enable a ‘wagon wheel’ approach to public debate.

## Conclusion

This article has demonstrated the enduring force of the paradigm of impartiality-as-balance. In the selected coverage we examined between 2007 and 2012, the BBC failed to change the paradigm – despite grand pronunciations in the Bridcut Report and good intentions carried over into the editorial policy. Although the research presented here has focused on the BBC, our second study which encompassed the other main terrestrial broadcasters, ITV and Channel 4, showed that this limiting operationalisation of impartiality is not unique to the BBC (Wahl-Jorgensen et al., 2013). The findings of this study, then, raise larger questions about the difficulties of representing the range of opinion in the context of public service broadcasting.

The consequences of the paradigm of impartiality-as-balance are manifold. First, it means that only a narrow range of views and voices are heard on the most contentious and important issues. Second, it results in reporting that focuses on party-political conflict, to the detriment of a journalism which provides much-needed context.

More than anything, our research demonstrates the power of professional paradigms – in this case, the paradigm of impartiality-as-balance. This may, of course, be particularly exacerbated by the traditional and long-standing two-party dominance of British politics, brought about by a first-past-the-post electoral system (e.g. Lipson, 1953). Nonetheless, the pattern of valuing an understanding of objectivity as balance holds true around the world (e.g. Berger, 2000), and is profoundly engrained in the everyday practice of journalists. There might be several reasons for the entrenched nature of this paradigm. Manning (2001) argues that the ‘pressure of news deadlines and the importance of obtaining information rich in news values, encourages a dependency upon official sources’ (p. 55). Also, journalists tend to ‘index’ the range of competing viewpoints to the intensity and duration of party-political conflicts (Bennett, 1994: 23). These practices have made it possible for journalism to capture and construct the news, and are unlikely to change unless the material circumstances of news production are profoundly transformed. But it does have profound consequences for the epistemology of journalism, insofar as the knowledge it produces is heavily structured by the reliance on opposing and polarised viewpoints, rather than a broader range of positions.

Paradigms within cultures of practice cannot be easily changed from the top-down, but rather must be transformed from the bottom-up. Such a shift is not easy to achieve in journalism, particularly not in the unique – and highly politically charged – context within which the BBC operates as a public service broadcaster. However, our research also suggests that some types of content – including online news – contain a broader range of news sources and views. This may point us to the ways in which the affordances of emerging technologies enable new discursive formations that may ultimately challenge the paradigm of impartiality-as-balance because they provide novel ways of thinking about what constitutes news and its sources.

## Notes

1. The content analysis was commissioned as part of the BBC Trust impartiality review and focused on assessing the breadth of opinion in the light of the association’s commitment to a greater diversity of views in its programming.

2. It should be noted that we opted to study up to 16 sources per story, as this captured the entirety of all sources cited in all but five of the stories analysed (the small number of exceptions included phone-in programmes). In cases where there were more than 16 sources, we coded only the first 16 sources mentioned.

3. We also analysed sources in terms of a range of demographic variables, including gender, age, ethnicity, religion and region, among many other things. Analysis of these has not been included in this article for reasons of space.

4. Online coverage on the relevant topics was identified by doing keyword searches during the sample period.

5. One variable (nationality of sources) was excluded from our reporting as a result of low intercoder reliability.

6. Please note that ‘national’ programming here refers to news programmes distributed across the United Kingdom on particular networks (BBC, ITV and Channel 4). In our report on the research, we refer to such programmes as ‘network’ programming to distinguish it from ‘nations and regions’ programming which is confined to particular nations and regions within the United Kingdom.

7. The more frequent appearance of the Scottish National Party in 2012 relates to stories around the party’s proposal for Scotland’s independence from the United Kingdom, and the consequences for EU membership that might result. This news event was very prominent in our online sample, but almost absent from television and radio coverage.

8. This statistic excludes the Today programme’s Thought for the Day feature, which skewed the sample given that it almost always used religious leaders as the main source.
